# Littoral cell angioma of the spleen: case report and literature review

**DOI:** 10.31744/einstein_journal/2024RC0267

**Published:** 2024-01-29

**Authors:** Paulo André Bispo Machado, Caroline de Oliveira Pereira, Julia Letícia de Bortolo, Ana Luísa Caetano Lopes Martins, Helder Groenwold Campos, Alan Junior de Aguiar, Rayana Pecharki Teixeira Alves Postai, Julio Cesar Wiederkehr, Henrique de Aguiar Wiederkehr

**Affiliations:** 1 General and Trauma Surgery Service Hospital do Trabalhador Universidade Federal do Paraná Curitiba PR Brazil General and Trauma Surgery Service , Hospital do Trabalhador , Universidade Federal do Paraná , Curitiba , PR , Brazil .; 2 Escola de Medicina Pontifícia Universidade Católica do Paraná Curitiba PR Brazil Escola de Medicina , Pontifícia Universidade Católica do Paraná , Curitiba , PR , Brazil .; 3 Digestive System Surgery Service Hospital Vita Batel Curitiba PR Brazil Digestive System Surgery Service , Hospital Vita Batel , Curitiba , PR , Brazil .

**Keywords:** Splenic neoplasms, Laparoscopy, Hemangioma, Splenectomy, Incidental findings, Tomography, X-ray computed

## Abstract

Littoral cell angioma is an extremely rare splenic vascular tumor originating from the cells lining the splenic red pulp sinuses. Approximately 150 cases of littoral cell angioma have been reported since 1991. Its clinical manifestation is usually asymptomatic and is mostly diagnosed as an incidental finding through abdominal imaging. Herein, we present a case of littoral cell angioma in a 41-year-old woman with no previous comorbidities, which initially presented as a nonspecific splenic lesion diagnosed on imaging in the emergency room. The patient was treated through laparoscopic intervention.

## INTRODUCTION

Primary splenic tumors are rare pathologies and can be classified as malignant or benign. Malignant tumors largely correspond to hemangiosarcomas and lymphomas. However, benign tumors encompass hemangiomas, hamartomas, hemangioendotheliomas, lymphangiomas, and littoral cell angioma (LCA). ^( 1 , 2 )^

Littoral cell angioma is an extremely rare splenic vascular tumor that originates from the lining cells of the splenic red pulp sinuses (littoral cells) with histiocytic and macrophagic characteristics, which are responsible for phagocytosing aged erythrocytes. ^( 3 )^ Histologically, LCA is defined as a well-delimited, nonencapsulated structure.

Its incidence is slightly higher in males, peaks in the fifth decade of life, and its diagnosis in children is extremely rare. ^( 4 )^ To date, approximately 150 cases of LCA have been reported since 1991, when Falk et al. described the first case of LCA. ^( 5 )^

Its clinical manifestation is usually asymptomatic, mostly diagnosed as an incidental finding on imaging. ^( 6 )^ When symptoms are present, the tumor usually shows nonspecific clinical features inherent to several other pathologies. Patients may present with splenomegaly, thrombocytopenia, anemia, weakness, fatigue, fever, and abdominal distension.

On imaging examinations, the lesions are mostly described as hypodense. They may be present in their multifocal form, with multiple splenic nodules of different sizes, or in their isolated form, as a single solitary nodule. ^( 7 )^ The definitive diagnosis is made through immunohistochemistry, making splenectomy a therapeutic and diagnostic approach. ^( 2 )^

Herein, we present a case of an LCA that initially presented as a nonspecific splenic lesion diagnosed on imaging in the emergency room and was successfully approached by laparoscopy.

## CASE REPORT

A 41-year-old woman with no previous comorbidities was admitted to the emergency room on July 15, 2021, complaining of persistent abdominal pain in the left hypochondrium radiating to the epigastrium, associated with nausea, for approximately 10 days. No other symptoms were observed. Vital signs revealed a temperature of 36.3 ºC, a respiratory rate of 22 breaths/min, a heart rate of 98 beats/min, and a blood pressure of 130/77mmHg.

Upon physical examination, diffuse pain in the left hypochondrium was noted during palpation, with no palpable abdominal masses or clinical signs of peritonitis. The results of the remaining physical examinations were normal. The laboratory test results are presented in table 1 . The patient reported a maternal history of Crohn’s disease, a mother with a history of lung cancer, and two uncles with a history of colon cancer.

**Table 1:** Admission laboratory tests

Blood count	-	Reference values
RDW	15.20%	11.50-14.50%
MCV	75.10fL	80.00-100.00fL
HCM	25.40pg	26.00-34.00pg
Creatinine	0.77mg/dL	0.66-1.09mg/dL
GFR	107mL/min/1.73 m ^2^	> 90.0mL/min/1.73 m ^2^
Urea	27.20mg/dL	17.0-43.0mg/dL
LDH	155U/L	208-378U/L
CRP	10.2mg/L	< 5.0mg/L
BT	0.33mg/dL	0.30-1.20mg/dL
BD	0.02mg/dL	<0.2mg/dL
BI	0.31mg/dL	<1.0mg/dL
AST	17U/L	<35U/L
ALT	18U/L	<35U/L
Calcium	8.9mg/dL	8.8-10.6mg/dL
Na+	140mEq/L	136-146mEq/L
K+	3.9mEq/L	3.5-5.1mEq/L
PU	Within normal parameters	

RDW: red cell distribution width; MCV: mean corpuscular volume; MCH: mean corpuscular hemoglobin; GFR: glomerular filtration rate; LDH: lactate dehydrogenase; CPR: C-reactive protein; BT: total bilirubin; BD: direct bilirubin; BI: indirect bilirubin; AST: aspartate aminotransferase; ALT: alanine aminotransferase; Ca: calcium; Na: sodium; K: potassium; PU: urine test.

**Figure 1:**
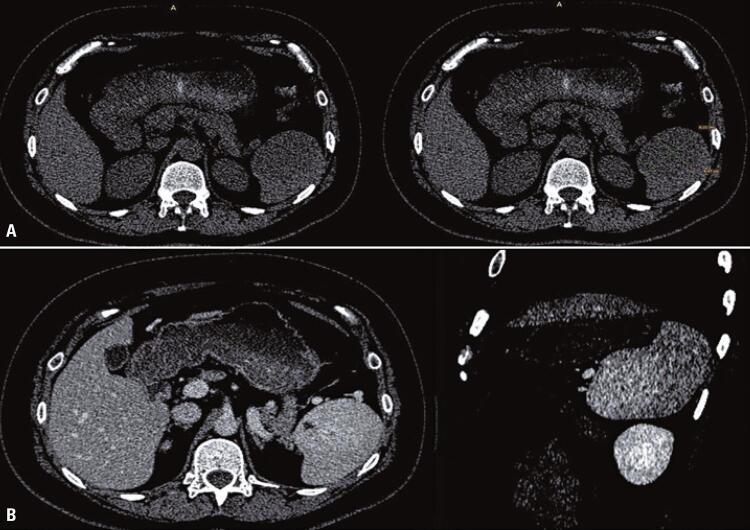
Computed tomography (without contrast) - Axial plane: oval, regular contoured, hypoattenuating mass located on the inferior and lateral aspect of the spleen (A). Computed tomography - Axial and sagittal plane: hypervascular enhancement about the adjacent splenic parenchyma, predominantly homogenous, presenting sparse hypovascular foci, without signs of extracapsular extension (B)

A computed tomography (CT) scan revealed a spleen of normal dimensions and a rounded, poorly delimited expansive mass measuring 49 × 43 × 49mm with heterogeneous enhancement after contrast ( Figure 1 ). No other lesions were observed in the solid organs of the abdomen or pelvis. During hospitalization, the patient underwent upper digestive endoscopy, which revealed mild pangastritis and Los Angeles grade A esophagitis, with no other commemorative findings. Based on the characteristics of the lesion and its symptoms, video laparoscopic splenectomy was performed.

**Figure 2:**
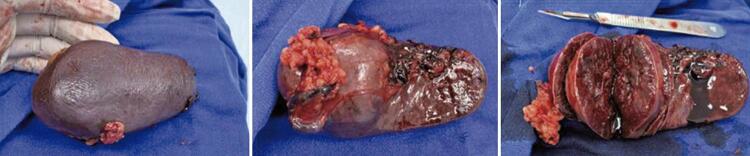
Macroscopic view of the spleen

During surgery, a lesion measuring approximately 4cm was observed between the spleen and tail of the pancreas, with a macroscopic heterogeneous, firm, brownish, and burgundy appearance ( Figure 2 ). An attempt to dissect the lesion to separate it from the pancreatic tail were unsuccessful; thus, the surgical team opted for splenectomy associated with caudal pancreatectomy.

The splenic artery (without enlarged lymph nodes) was sectioned using Hemolok clips. An exophytic lesion intensely adhering to the pancreatic tail without a cleavage plane was identified, and the pancreas was sectioned with a stapler using bioabsorbable staple line reinforcement. The splenic vein was dissected and sectioned near the junction with the superior mesenteric vein using HemoLok clips. The distal pancreas was lifted, and the resection plane included the retroperitoneal space. The short gastric veins were divided, and the distal pancreas was removed in a block with the spleen.

The surgical specimen was placed in a plastic bag and removed using a Pfannenstiel incision. A tubule-laminar drain (Blake ^®^ ) was also used to monitor the pancreatic stump, which evolved with negligible output on the third postoperative day. The macroscopic pathological study described a spleen of 163g and 15.5 x 5.8 x 4.4cm, with the presence of an expansive nodular lesion, not encapsulated, measuring 5.9 x 4.5 x 3.9cm, under the splenic capsule, without perforation, and with adherence to the adipose tissue. The adjacent tissues showed no abnormalities.

Immunohistochemistry revealed a splenic tumor with positive immunohistochemical profiles of CD68+, CD34+, and CD31+ cells. The patient was followed up in the outpatient clinic, with a new CT performed after 3 months showing no significant alterations, and was subjected to *pneumococcus, meningococcus* , and Haemophilus vaccination. Owing to the common association of LCA with other neoplasms, colonoscopy and upper digestive endoscopy examinations were also performed for tracking purposes and showed no alterations. After 10 months, the patient remained asymptomatic, with amelioration of the abdominal pain and no other gastrointestinal symptoms.

The study was approved by the Research Ethics Committee of *Pontifícia Universidade Católica do Paraná* (CAAE: 62968222.4.0000.0020; # 5.657.922).

## DISCUSSION

Primary vascular neoplasms of the spleen are rare entities classified into three large groups based on their histological features: tumors with conventional endothelium, lymphatic endothelium, or specialized endothelium. ^( 8 )^ Littoral cell angioma is a rare benign spleen tumor that originates from the cells lining the splenic pulp and is composed of differentiated endothelial and histiocytic tissues. ^( 9 )^

The incidence of the disease varies from 1-77 years and is more prevalent among middle-aged people, without a significant gender difference. In a retrospective study involving 27 patients with LCA, Cai et al. reported a median age of 45 years, and only two children were diagnosed with the disease. ^( 10 )^

Clinically, LCA may present asymptomatically or with constitutional and nonspecific symptoms such as abdominal pain, fever, anorexia, and occasionally splenomegaly on physical examination. Owing to the absence of specific clinical signs for LCA, this tumor is usually diagnosed during the investigation of anemia or thrombocytopenia in laboratory findings or can be found as an accidental mass in abdominal imaging tests. ^( 6 )^

In the present case, the patient fell within the expected age range based on previous studies, and the main complaint was diffuse abdominal pain without other specific gastrointestinal symptoms. The patient did not present with palpable abdominal masses during the physical examination; only the CT image was suggestive of a mass in the spleen, showing a rounded and poorly defined expansive lesion with heterogeneous enhancement after contrast.

Imaging tests are the main method of initial investigation of LCA; however, contrast between CT and magnetic resonance imaging (MRI) is ineffective in differentiating this tumor from other angiomas, angiosarcomas, lymphomas, or splenic metastases. ^( 11 )^ Usually, LCA is present in a multifocal manner. Contrast-enhanced CT can vary from isodense to hypodense lesions related to the surrounding splenic parenchyma and is generally well delimited. Similarly, a T1-weighted MRI image showed a hypointense mass, whereas in the T2, the lesion was hyperintense. ^( 9 )^ Despite the lack of specificity, visualization of the mass in the splenic territory during CT or MRI rules out certain differential diagnoses. It determines the extent of the disease, which is an important attribute in a possible pre-operative program.

**Figure 3:**
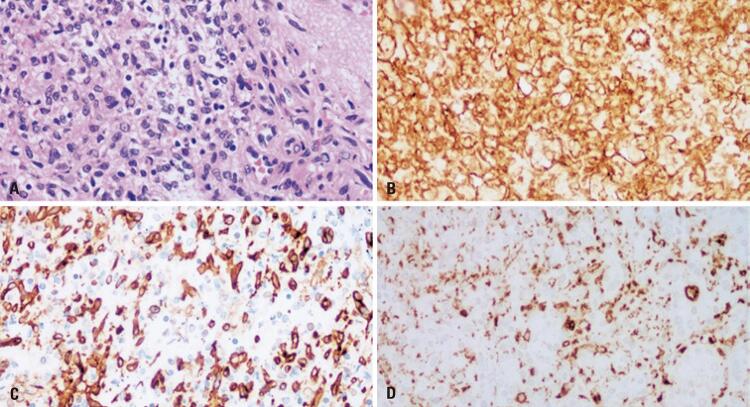
High magnification view shows the characteristic histologic appearance of the tumor. (A) Vascular spaces lined by plump endothelial cells (H&E stain, x40); (B) Endothelial marker CD31 (CD31 stain, x40); (C) Endothelial marker CD34 (CD34 stain, x40); (D) Endothelial marker CD68 (CD 68 stain, x40)

A biopsy is used to identify the specific characteristics of LCA and is usually associated with a combined pattern of endothelial and histiocytic cells on histology, as immunohistochemical (IHC) markers are positive for CD31+, factor VIII+, and CD68+ ( Figure 3 ). ^( 4 , 12 , 13 )^ Usually, LCA does not express the CD8 marker because it is found only in normal littoral cells in which the architecture of the splenic red pulp is preserved, as well as CD34. The presence of infiltrative spindle cells on IHC in LCA suggests malignant potential with the possibility of metastasis, and long-term surveillance may be required. ^( 8 )^ Other studies have shown the possibility of malignancy, ^( 14 )^ especially if the organ weighs >1500g or sizes >20cm ^2^ . ^( 11 )^ In this case, the spleen weighed 163g, and microscopic studies indicated a benign pathology.

In most cases, analysis of the splenic tissue occurs only postoperatively, as cytology through fine-needle puncture confers a high risk of bleeding. In the case of a malignant neoplasm, it can cause dissemination. ^( 15 )^

One of the reasons for the importance of describing an LCA case is that this tumor may be associated with other visceral neoplasms in up to 60% of the two cases, ^( 9 )^ which may include renal, hepatic, hematological, and gastrointestinal tumors. ^( 16 , 17 )^ In a series of cases conducted by Peckova et al., 25 patients with LCA were followed up for approximately 11 years, and 15 patients were diagnosed with other tumors during follow-up. ^( 13 )^

In addition, the LCA may present as a metastatic tumor. Opatrny et al. initially diagnosed a splenic tumor as a suspected metastatic tumor during the investigation of adenocarcinoma of the colon; however, IHC was compatible with LCA. ^( 9 )^ Similarly, Cosme et al. also discovered LCA after a colonoscopy examination that showed a polyp in an asymptomatic patient through investigation with abdominal CT for metastasis screening. ^( 18 )^

Thus, it is recommended that patients diagnosed with LCA be screened for other neoplasms to offer early intervention and make a differential diagnosis between LCA and metastasis from other sites. In our case, no association with other neoplasms was found because upper gastrointestinal endoscopy and colonoscopy examinations after surgery were negative for other tumors, in addition to a new CT of the lungs and abdomen with no alterations. In our patient, the only risk factor for LCA was a family history of first-grade inflammatory bowel disease.

Littoral cell angioma is considered a benign tumor of the spleen; however, its removal is always indicated, whether using a conventional or laparoscopic approach. ^( 3 )^ The surgical indications include the possibility of anemia and/or pancytopenia, malignancy risk, and possible rupture due to excessive organ enlargement. Two previous reports have described the clinical manifestations of LCA with splenic rupture and hemoperitoneum. ^( 19 , 20 )^ In our study, we opted for a laparoscopic approach, which offers a shorter surgical time and minimal blood loss, as observed by other authors. ^( 10 )^

## CONCLUSION

The rarity of littoral cell angioma remains a diagnostic challenge in clinical practice. Despite being considered a benign tumor in most cases, it may be related to other neoplasms. In this case, the clinical manifestations were nonspecific, with a diagnosis made by immunohistochemistry, and the laparoscopic approach was effective.
